# Clinical and genetic determinants of glioblastoma survival: a retrospective study

**DOI:** 10.3389/fnmol.2026.1740199

**Published:** 2026-02-26

**Authors:** Julia L. Gutiérrez-Arroyo, Pia Gallego-Porcar, Elvira Carbonell-Martinez, Luis G. González-Bonet, Maria Victoria Ibañez, María Díaz-Ruiz, Hugo Caballero-Arzapalo, Ariadna Soto, Guillermo Garcia-Oriola, Jose Maria Borras-Moreno, Conrado Martinez-Cadenas, Maria Angeles Marques-Torrejon

**Affiliations:** 1 Department of Neurosurgery, Castellon General University Hospital, Castellón de la Plana, Spain; 2 Department of Medicine, Jaume I University of Castellon, Castellón de la Plana, Spain; 3 Department of Mathematics, Jaume I University of Castellon, Castellón de la Plana, Spain; 4 Clinical Laboratory, Castellon General University Hospital, Castellón de la Plana, Spain

**Keywords:** genetic mutations, glioblastoma survival analysis, prognosis, tumor features, ventricular system

## Abstract

**Background:**

This study analyzed 57 patients with glioblastoma treated at the General University Hospital of Castellon, Spain, focusing on clinical, tumor-specific and genetic factors influencing disease outcome. Variables included age, sex, BMI, extent of surgical resection, and use of radiotherapy or chemotherapy. Tumor characteristics assessed included location, size, proximity to the ventricular system and surgical approach. Genetic mutations in the *EGFR*, *TP53* and *CDKN2A* genes were also analyzed.

**Methods:**

Kaplan–Meier survival analysis was used to assess the impact of clinical, tumor-related, treatment, lifestyle and genetic variables on overall survival and progression-free survival, with group differences evaluated using log-rank tests. Given the exploratory nature of the study and the sample size, multivariable modeling was not performed. Patients with IDH1/2-mutant tumors were excluded in accordance with the 2021 World Health Organization (WHO) classification, which no longer defines IDH-mutant grade 4 astrocytomas as glioblastoma.

**Results:**

A significant finding was the strong association between extent of resection, tumor proximity to the ventricular system and survival: patients with tumors closer to the ventricles had significantly shorter survival, highlighting the critical role of spatial tumor characteristics in glioblastoma outcomes.

**Conclusion:**

These results suggest that integrating clinical, genetic and spatial tumor data into personalized treatment approaches could improve prognosis.

## Highlights

-Total tumor resection and absence of contact with the ventricles were associated with longer survival.
-Age, sex, std treatments, genetic mutations, and secondary surgeries showed no effect on survival.

## Introduction

Glioblastoma (GB), classified as a grade 4 astrocytoma by the World Health Organization (WHO) (Louis et al., 2021; Obrador et al., 2024), is the most prevalent, aggressive, and lethal primary brain tumor in adults. Known for its rapid growth, high invasiveness, and significant molecular diversity (Louis et al., 2021), it typically arises in the cerebral hemispheres (Price et al., 2024; Thakkar et al., 2014). Despite advances in treatment, including extensive surgical resection, radiotherapy, and temozolomide-based (Valenzuela-Fuenzalida et al., 2024) chemotherapy (Stupp protocol) (Stupp et al., 2005), the median survival remains dismally poor at 12–15 months post-diagnosis. This reflects the urgent need for novel therapies that effectively target the biological and molecular complexity (Davis, 2016; Vehlow and Cordes, 2013).

Glioblastoma exhibits extensive heterogeneity and plasticity at the cytopathological, transcriptional, and genetic levels (Becker et al., 2021; Perrin et al., 2019; Brennan et al., 2013; Kim et al., 2015). Moreover, the blood–brain barrier (BBB) and altered tumor permeability represent additional major obstacles to effective therapy, further limiting drug delivery and treatment efficacy (Ahmed et al., 2023; Davis, 2016; Wu et al., 2021). Within this highly complex microenvironment, glioblastoma stem-like cells (GSCs) have emerged as key drivers of tumor progression, therapeutic resistance, and recurrence (Loras et al., 2023; Stiles and Rowitch, 2008; Safa et al., 2015; Xie et al., 2022; Hu et al., 2023; Singh et al., 2004). GSCs exhibit marked resistance to apoptosis, contribute to angiogenesis and immunosuppression, and play a central role in both radio- and chemo-resistance (Di Nunno et al., 2022; Schaff and Mellinghoff, 2023; Furnari et al., 2007; Atkins et al., 2015). These properties are further reinforced by hypoxic conditions within the tumor microenvironment, which promote the maintenance of stem-like phenotypes and enhance tumor adaptability (Yalamarty et al., 2023; Eckerdt and Platanias, 2023). The persistence of therapy-resistant GSC populations is widely regarded as a major reason why glioblastoma almost invariably recurs following standard treatment (Bao et al., 2006).

Recent advances in genomic profiling have provided deeper insights into the genetic alterations driving GB. *IDH1*/*2* mutations are predominantly found in grade 4 astrocytomas that evolve from lower-grade gliomas (secondary GBs) (Ichimura et al., 2009), which differ molecularly and clinically from primary GBs (Ichimura et al., 2009). Secondary GBs are different from primary GBs because they develop from pre-existing lower-grade gliomas (classified as WHO grades 2 or 3) instead of appearing out of nowhere, like primary GBs do (Delgado-Martín and Medina, 2020). Lower-grade gliomas tend to grow more slowly and have better survival rates initially, but over time, they can pick up additional genetic changes, such as mutations in *TP53* or loss of *ATRX*, which can eventually lead to their progression into secondary GBs (Claus et al., 2015). This distinction between primary and secondary GBs is crucial because it highlights the differences in their molecular and clinical pathways (Mao et al., 2012). In contrast, *EGFR* amplification, present in approximately 40% of GB cases, is linked to worse outcomes by promoting aggressive tumor behavior through constitutive activation of growth signaling pathways (Verhaak et al., 2010). *TP53* mutations, affecting the p53 pathway in 87% of cases, significantly contribute to disease progression, while alterations in *CDKN2A*, a key cell cycle regulator gene, further impair apoptosis and disrupt cell cycle control (Campbell et al., 2016). *RTK/PI3K/PTEN* pathway alterations are observed in 88% of GB cases, emphasizing their role in tumourigenesis. Additionally, loss of heterozygosity on chromosome 10 is one of the most common chromosomal abnormalities identified (Fujisawa et al., 2000). These genetic insights not only enhance our understanding of GB biology but also have potential to improve diagnosis, predict outcomes, and inform personalized therapies. For example, *IDH* mutations offer prognostic value, while *EGFR*, *TP53*, and *CDKN2A* alterations highlight the molecular complexity and therapeutic resistance of GB (Dunn et al., 2012).

In addition to genetic drivers, clinical and lifestyle factors such as age, sex, overall health, smoking, family history, and viral infections may influence prognosis, though their roles in GB remain underexplored. Some studies suggest sex-based survival differences, with longer survival in females (Yang et al., 2013). Lifestyle factors such as smoking, family cancer history, and viral infections (e.g., hepatitis B, COVID-19) may also influence disease progression and treatment response, although their roles in GB remain underexplored (Travers and Litofsky, 2021).

Tumor location relative to brain ventricles has emerged as a critical prognostic factor, though evidence in mixed impact on overall survival (OS) have been inconsistent (Mistry et al., 2020). While GB distance from the subventricular neural stem cell niche has not been correlated with survival (Mistry et al., 2017), a recent meta-analysis reported that GBs in contact with the lateral ventricle are associated with lower survival. This effect may be independent of established survival predictors, emphasizing the clinical relevance of the ventricular-subventricular zone contact in GB biology (Mistry et al., 2017; Travers and Litofsky, 2021). Furthermore, tumor location near the third ventricle and the contrast-enhancing tumor border has been identified as a prognostic factor, particularly in elderly patients (Fyllingen et al., 2021). Understanding these spatial relationships and their biological implications is crucial for devising more effective therapies and improving GB prognosis.

This study aimed to evaluate clinical, surgical and genetic determinants of survival in glioblastoma, with particular emphasis on the spatial relationship between tumor and ventricle.

## Methods

### Study design and patient selection

This retrospective cohort study includes 57 adult patients diagnosed with GB at the Castellon General University Hospital, Castellon, Spain. The recruitment period was from January 2020 to August 2023. Patients were selected based on confirmed GB diagnosis and the availability of complete clinical and genetic data. All results reflect data up to February 12th, 2023, with patient survival information updated to this date.

The study was approved by the Drug Research Ethics Committee (CEIm) of the General Hospital University of Castellon, Spain. In accordance with the 2021 WHO classification of tumors of the central nervous system, patients harboring IDH1 or IDH2 mutations were excluded to ensure a homogeneous cohort of IDH-wildtype glioblastoma.

Baseline clinical, tumor-related, treatment, lifestyle, and genetic characteristics of the cohort are summarized in Table 1.

**TABLE 1:** Baseline clinical, tumor-related, treatment, lifestyle, and genetic characteristics of the glioblastoma cohort.

Variable	Total
*n*	57
Age, years, median (range)	64 (17–81)
**Sex, *n* (%)**	
Female	27 (47.4%)
Male	30 (52.6%)
BMI, median (IQR)	25.35 (IQR 24.22–28.57)
**Tumor–ventricle distance group, *n* (%)**	
Close (T1)	19 (33.3%)
Intermediate (T2)	19 (33.3%)
Far (T3)	19 (33.3%)
Tumor volume (cm^3^), median (IQR)	9,216 (IQR 3,600–32,164)
**Extent of resection, *n* (%)**	
Biopsy	18 (31.6%)
Partial resection	26 (45.6%)
Total resection	13 (22.8%)
**Radiotherapy, *n* (%)**	
No	5 (8.8%)
Yes	52 (91.2%)
**Chemotherapy (TMZ STUPP), *n* (%)**	
No	8 (14.0%)
Yes	49 (86.0%)
**Death (exitus), *n* (%)**	
Yes	23 (41.1%)
No	33 (58.9%)
*Recurrence, *n* (%)**	23 (40.4%)
**Smoking status, *n* (%)**	
No	30 (52.6%)
Yes	27 (47.4%)
**Family history of cancer, *n* (%)**	
No	36 (63.2%)
Yes	21 (36.8%)
**Hepatitis B, *n* (%)**	
No	17 (29.8%)
Yes	40 (70.2%)
**Type 2 diabetes, *n* (%)**	
No	27 (47.4%)
Yes	30 (52.6%)
**EGFR alteration, *n* (%)**	
Yes	15 (26.3%)
No	42 (73.7%)
**TP53 alteration, *n* (%)**	
Yes	17 (29.8%)
No	40 (70.2%)
**CDKN2A alteration, *n* (%)**	
Yes	20 (35.1%)
No	37 (64.9%)

Summary of demographic variables, tumor features, treatment modalities, comorbidities, and genetic alterations for the 57 patients included in the study. Continuous variables are reported as median (range or interquartile range, IQR), and categorical variables are shown as number (percentage). Tumor–ventricle distance groups were defined using cohort-specific tertiles (T1: close, T2: intermediate, T3: far). Genetic alterations refer to the presence of somatic mutations or copy number variations detected by targeted next-generation sequencing. *Recurrence data are based on available follow-up during the study period and may be affected by censoring. **Percentages may not sum to 100% due to rounding.

### Data collection

For each patient, we created a database including different factors (Supplementary material):

**1. Demographic and Clinical Variables**: sex, age at diagnosis, year of birth, BMI (calculated from weight and height), and survival metrics (OS, PFS).

**2. Tumor and Treatment Details**: tumor location (frontal, parietal, temporal, occipital), tumor subtype, and treatment information including radiotherapy, extent of surgical resection (total, partial or subtotal, biopsy), and chemotherapy regimen (Stupp protocol with temozolomide, PCV regimen, and adjunctive use of Bevacizumab). In addition, the distance between the tumor and the ventricular system was quantified on preoperative contrast-enhanced MRI. The coronal slice showing the maximum tumor extension was selected, and the minimum linear distance between the enhancing tumor margin and the ependymal surface of the lateral ventricle was measured. To ensure consistency and reproducibility, measurements were performed using a standardized anatomical approach. For statistical analyses, tumor–ventricle distance was stratified into three groups based on tertiles of the observed distance distribution to ensure balanced group sizes. Patients were assigned to the closest tertile (T1, “close”), the intermediate tertile (T2, “intermediate”), or the farthest tertile (T3, “far”), according to increasing tumor–ventricle distance. In this cohort, these tertiles corresponded to distances of 0–5.20 mm (T1), 5.2–12.45 mm (T2), and 12.6–41.20 mm (T3); group assignment was based on distribution tertiles rather than predefined absolute distance thresholds.

Extent of resection was determined from early postoperative contrast-enhanced MRI performed within 24 h after surgery, complemented by surgical reports, in order to avoid misinterpretation with postoperative changes. This information was available for all 57 patients.

**3. Recurrence Data:** the recurrence type, date, time to recurrence, and details of any secondary surgical intervention were collected. Additionally, the distance from the tumor to the nearest ventricle.

Tumor volume was determined preoperatively on contrast-enhanced T1-weighted images, segmenting the entire enhancing lesion. Measurements were confirmed with a neuronavigation system (StealthStation™ S8, Medtronic).

Chemotherapy: the majority of patients received concomitant temozolomide radiotherapy and maintenance temozolomide (EORTC/NCIC regimen, often referred to as the Stupp protocol). Dosing followed standard schedules: 75 mg/m^2^/day during 42 days of RT, followed by adjuvant 150–200 mg/m^2^/day for 5 days every 28 days, up to 6 cycles. A minority of patients received PCV chemotherapy or adjuvant bevacizumab at physician discretion in recurrent/high-risk cases.

**4. Lifestyle and Medical History**: presence of prior cancer, smoking status, history of COVID-19 infection, hepatitis B status, and Type II diabetes. The inclusion of variables such as diabetes or hepatitis B was exploratory, based on previous reports suggesting potential prognostic roles of systemic comorbidities in glioblastoma.

**5. Genetic Analysis:** NGS was performed in tumor DNA and tumor RNA on a panel of target genes. DNA and RNA extraction was performed automatically using QiAcube extractor (Qiagen, Hilden, Germany) following the manufacturer’s instructions. Massive NGS sequencing was performed with Ion Torrent technology (Ion Torrent™ Genexus™ Integrated Sequencer) from Thermo Fisher Scientific (Waltham, MA, USA). The Oncomine Precision panel - GX5 - Solid Tumor - w3.2.0 DNA and Fusions Panel was used with target regions defined in Target Regions Oncomine precision v3.6.20210407.designed.bed. The genetic sequencing data was analyzed at the Department of Clinical Analysis of the Castellon General University Hospital, following their standard protocol for post-surgery tumor examination.

The bioinformatics platform used was Genexus System. The detected variants have been filtered and visualized with the Ion Reporter Software 5.18 and IGV Integrative Genomics Viewer programs. The detection limit of the technique is VAF > 0.5%. The cancer panel sequenced included the following genes, where two types of genetic mutations were determined: (i) cancer driving mutations in the following genes: *IDH1*, *IDH2*, *EGFR*, *TP53*, *KRAS*, *HRAS*, *RET*, *PTEN*, *NTRK1*, *PIK3CA*, *MAP2K1*, and *BRAF*; and (ii) copy number variations (CNVs) in the following genes *EGFR*, *CDKN2A*, *FGFR2*, *PTEN*, *AR*.

### Statistical analysis

Statistical analyses were performed using SPSS and R (RStudio). Kaplan–Meier survival analysis was performed to assess overall survival (OS) and progression-free survival (PFS) in relation to clinical and treatment variables [sex, radiotherapy, chemotherapy, surgical type (total, partial, biopsy), tumor location, tumor–ventricle distance, and tumor size] as well as genetic factors (presence of somatic mutations in *IDH*, *EGFR*, *TP53*, and *CDKN2A*). Survival differences between groups were evaluated using log-rank tests.

Progression-free survival (PFS) was defined as the time from surgery to radiological progression on follow-up MRI examinations (1 month, 3 months, and every 6 months thereafter).

Statistical analyses were conducted in RStudio. Non-parametric tests (Kruskal–Wallis and pairwise Wilcoxon tests with Bonferroni correction) were applied to evaluate associations between tumor–ventricle distance, extent of resection, and tumor volume. A two-sided *p*-value < 0.05 was considered statistically significant. Data visualization was performed using the ggplot2 and ggpubr packages.

To assess the relationship between tumor volume analyzed in MRI images and overall survival, we first performed a linear regression analysis using tumor volume (cm^3^) as the independent variable and survival in days as the dependent variable. As linear regression does not account for censoring, this analysis was considered exploratory and descriptive only; primary time-to-event comparisons relied on Kaplan–Meier and log-rank tests. A scatter plot with a fitted linear model was generated using the ggplot2 package in R. Tumor volume was also categorized into three groups (small, medium, large) divided in quartiles for comparison of survival distributions also using the ggplot2 package in R.

## Results

Univariate survival and exploratory analyses of clinical, tumor-related, genetic, and lifestyle variables are summarized in Table 2. Overall survival was primarily influenced by tumor proximity to the ventricular system, whereas no significant associations were observed for demographic, treatment-related, genetic, or lifestyle variables.

**TABLE 2:** Summary of univariate survival and exploratory statistical analyses performed in the glioblastoma cohort.

Variable	Groups (*n*)	Statistical test	*P*-value (nominal)	FDR (BH)	Interpretation
Sex	Male (30) vs. Female (27)	Log-rank	0.36	0.68	No nominal association with OS
Age	Continuous (descriptive)	Descriptive analysis	NA	NA	Age distribution was comparable across patients
BMI	Continuous (descriptive)	Descriptive analysis	NA	NA	No evident association with OS
Radiotherapy	Yes (55) vs. No (2)	Log-rank	0.185	0.59	No nominal association with OS
Chemotherapy	Yes (56) vs. No (1)	Log-rank	0.94	0.96	No nominal association with OS
Extent of resection	Total (13) / Partial (28) / Biopsy (16)	Log-rank	0.079	0.59	Nominal trend toward improved OS with total resection
Tumor location	Frontal / Temporal / Parietal / Occipital	Log-rank	0.31	0.68	No nominal association with OS
Tumor–ventricle distance	T1 close / T2 middle / T3 far	Log-rank	**0.0012**	**0.0192**	Nominal association with OS; reduced survival observed in tumors closest to the ventricle
Tumor volume (OS)	Tertiles (19 / 19 / 19)	Log-rank	0.42	0.68	No nominal differences in OS
Tumor volume vs. OS	Continuous	Linear regression	0.85	0.96	No evidence of correlation with OS
Tumor volume vs. ventricle distance	Continuous	Linear regression	0.53	0.68	No evidence of association
EGFR mutation	Mutated (12) vs. WT (45)	Log-rank	0.55	0.73	No nominal association with OS
TP53 mutation	Mutated (18) vs. WT (39)	Log-rank	0.47	0.68	No nominal association with OS
CDKN2A alteration	Altered (21) vs. WT (36)	Log-rank	0.66	0.81	No nominal association with OS
Family cancer history	Yes (23) vs. No (34)	Log-rank	0.32	0.68	No nominal association with OS
Smoking status	Smoker (26) vs. Non-smoker (31)	Log-rank	0.82	0.82	No nominal association with OS
Hepatitis B	Positive (41) vs. Negative (16)	Log-rank	0.39	0.68	No nominal association with OS
Type 2 diabetes	Yes (31) vs. No (26)	Log-rank	0.18	0.59	No nominal association with OS
COVID-19 history	Yes (31) vs. No (26)	Log-rank	0.44	0.68	No nominal association with OS
Recurrence	Yes (22) vs. No (35)	Log-rank	>0.05	NA	No nominal association with OS
Secondary surgery	Yes (12) vs. No (45)	Log-rank	>0.05	NA	No nominal association with OS

Overall survival (OS) was evaluated using Kaplan–Meier survival curves and compared between groups using log-rank tests. Associations between continuous variables and survival or tumor characteristics were explored using descriptive analyses or linear regression, as appropriate. Non-parametric tests were applied where indicated. A two-sided *p*-value < 0.05 was considered statistically significant. Bold values indicate statistically significant results (*p* < 0.05).

### Demographic and clinical variables

In this study, we looked at 57 patients who were diagnosed with GB. The median age at the time of diagnosis was 61.5 years, with ages ranging from 16 to 80 years. Interestingly, there was an almost even split between male and female patients, with a 1:1 ratio. The average BMI (Body Mass Index) of the group was 26.6 kg/m^2^, with a standard deviation of ±4.2. When it came to survival, the median OS was 13.7 months, ranging from as little as 3 months to as long as 32 months. The median progression-free survival (PFS) was slightly longer, at 12.4 months, with a range of 1–18 months.

**FIGURE 1:**
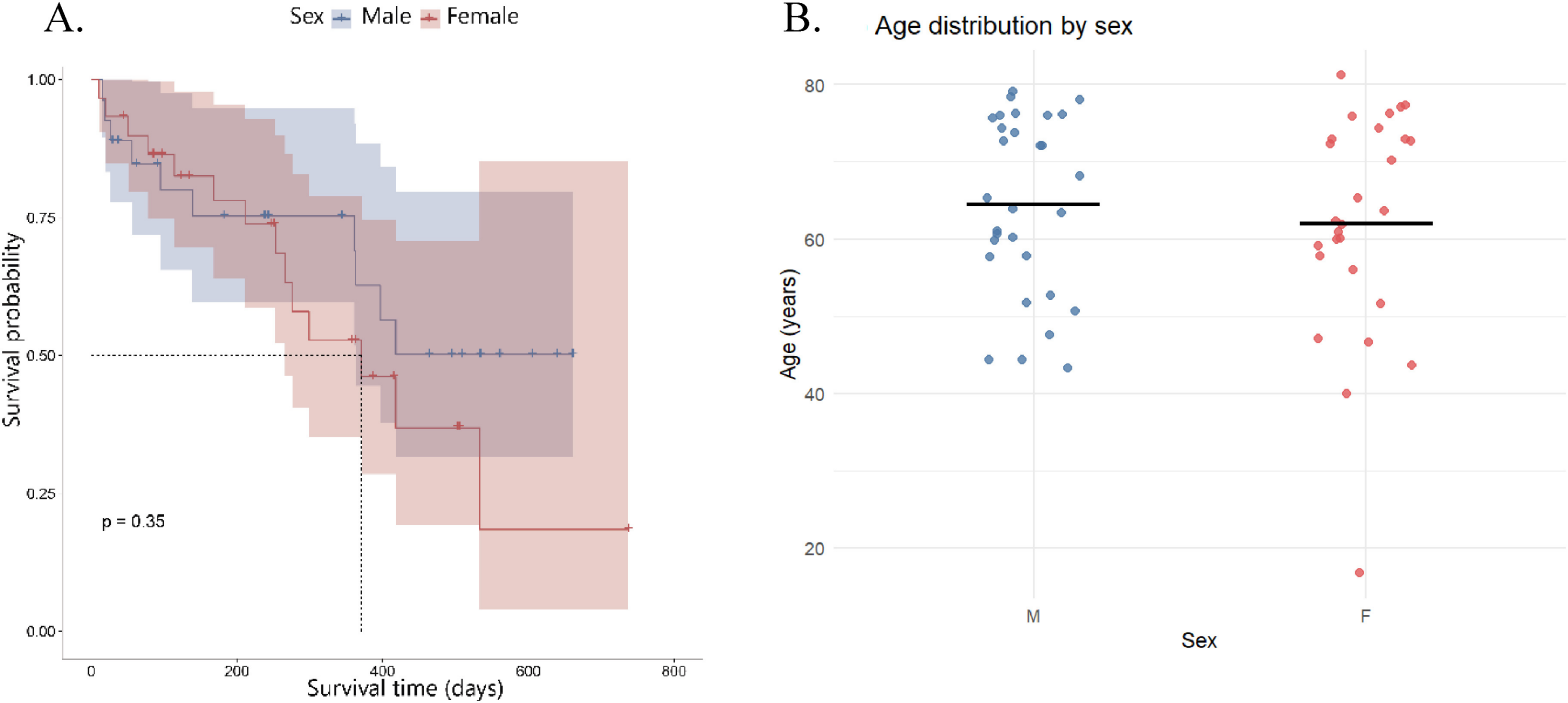
Survival and cohort heterogeneity by sex. **(A)** Kaplan–Meier overall survival (OS) curves for glioblastoma patients stratified by sex: female patients (pink line) and male patients (blue line). **(B)** Distribution of patient age stratified by sex. Each point represents an individual patient, with horizontal bars indicating the median age. Male and female patients show comparable age distributions, illustrating the demographic heterogeneity of the cohort without evident sex-related differences.

Using Kaplan-Meier survival curve, we also analyzed whether demographic factors like sex and age (Figure 1A), or BMI had any impact on survival. From this analysis, no significant differences in survival were observed between sexes (*p* = 0.36). Figure 1B depicts the age distribution of the patient cohort stratified by sex. Individual data points and median values indicate a largely overlapping age range between male and female patients, suggesting comparable baseline age distributions and supporting the absence of marked sex-related differences in this cohort of distinct clustering by these variables.

### Tumor features and treatment details

**FIGURE 2:**
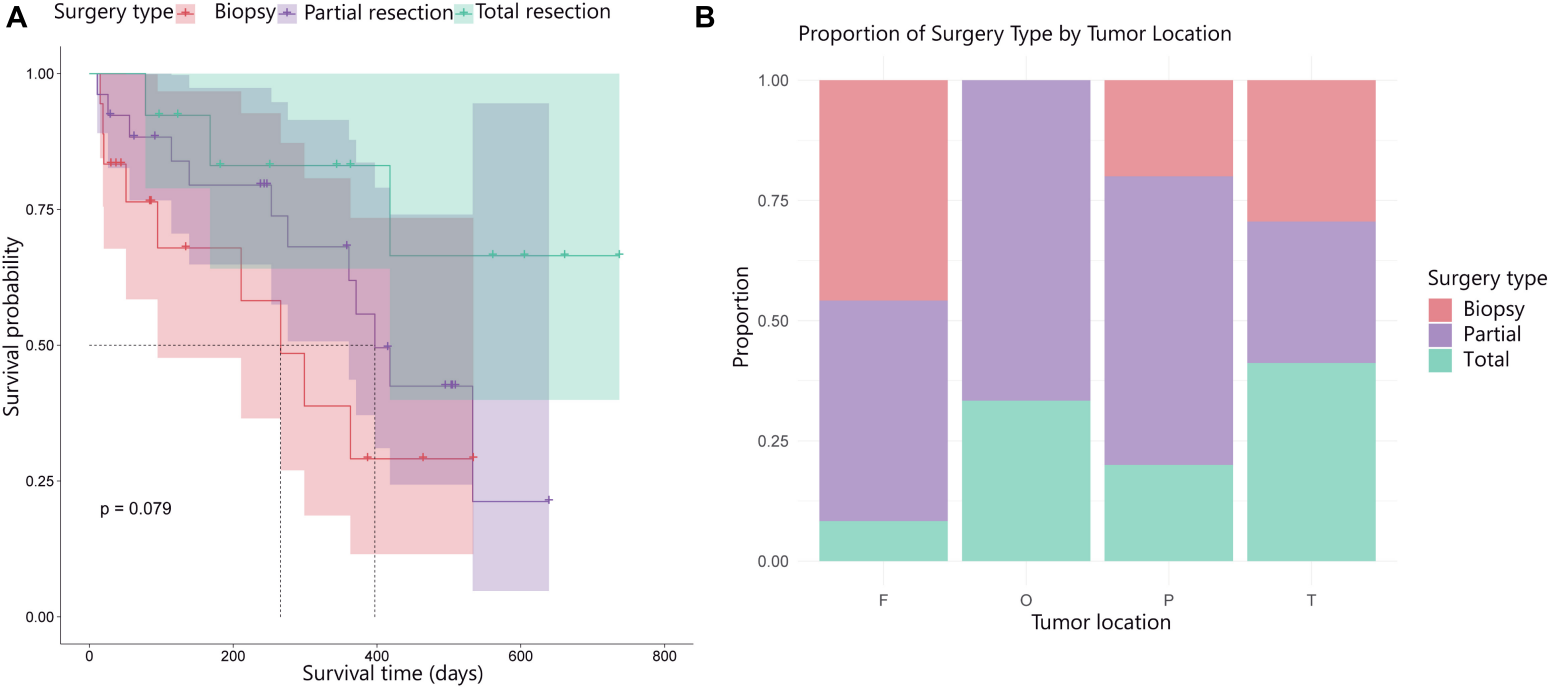
Survival and tumor location related to type of surgery. **(A)** Kaplan–Meier survival curves according to surgical procedure (biopsy, partial resection, or total resection). A trend toward longer survival was observed for gross total resection compared to partial resection and biopsy (log-rank *p* = 0.079). **(B)** Distribution of tumor locations (F, frontal; T, temporal; P, parietal; O, occipital) across the cohort, showing that most lesions were frontal and temporal.

Tumor locations in this study varied, but most were found in the frontal and temporal lobes (Figure 2B). Surgical resection emerged as a key factor influencing OS. Nearly all patients received standard radiotherapy (*n* = 55) and chemotherapy (*n* = 56, mostly concomitant temozolomide radiotherapy and maintenance temozolomide). When stratifying survival by type of surgery, a trend toward improved outcomes was observed (Figure 2B). Patients who underwent gross total resection showed longer OS, whereas those with subtotal resection had shorter survival, and patients who only underwent biopsy had the poorest outcomes. Although the log-rank test did not reach statistical significance (*p* = 0.079), the pattern suggests a survival advantage for patients undergoing more extensive resections.

We next examined whether tumor location affected the surgical approach. Frontal and parietal tumors were mainly treated with partial resection or biopsy, while temporal lesions more often allowed total resection. Overall, tumor location appeared to influence the extent of resection, which may partly explain survival variability among patients.

### Effect of recurrence, tumor location and distance of the tumor to the ventricle on overall survival

Out of all 57 patients included in the study, tumor recurrence was documented in 25 out of 57 patients (43.9%) during follow-up during the study period. The median time to recurrence was relatively short, at 3.4, indicating that recurrence was frequent and occurred early despite standard treatment. Secondary surgeries were performed in 12 patients; however, these additional procedures did not result in a significant improvement in overall survival, highlighting that re-intervention alone was insufficient to overcome tumor progression.

When tumor location (Figure 2A) in the brain was analyzed in relation to OS, no meaningful correlations were found. This suggests that survival outcomes are not strongly influenced by the specific lobe or region of the brain where the tumor was located.

**FIGURE 3:**
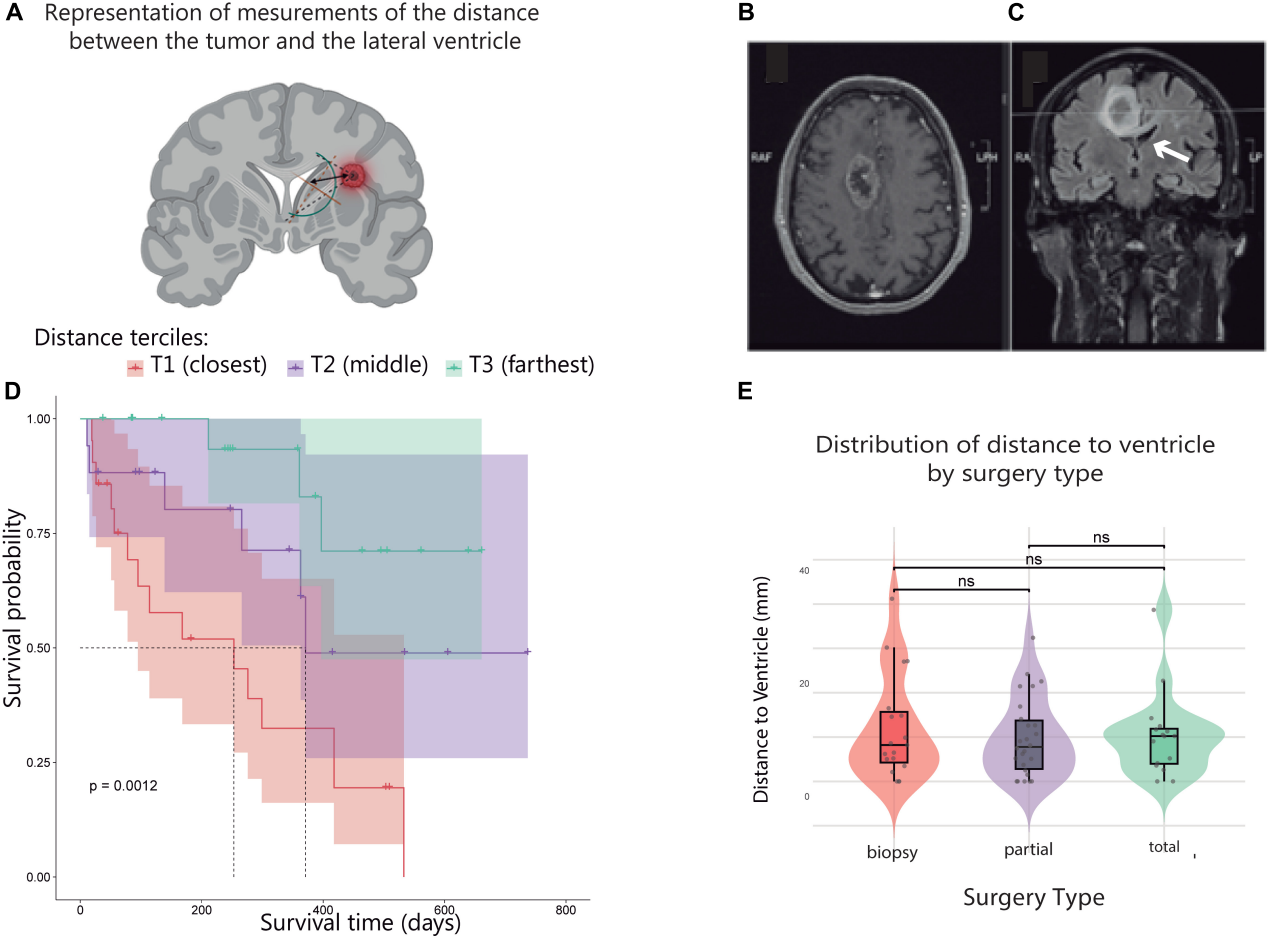
The distance to the lateral ventricle in relation to the type of surgery and survival. **(A)** Schematic representation of the standardized measurement of the minimum distance between the tumor border and the lateral ventricle on coronal MRI sections. Arrows indicate vectors from the closest tumor edge to the ventricular ependyma, which were used to classify samples based on their proximity to the ventricular system. **(B)** fMRI scan showing glioblastoma (GB) location relative to the lateral ventricle in axial view, highlighting tumor proximity to ventricular structures. **(C)** fMRI scan showing glioblastoma (GB) location relative to the lateral ventricle in coronal view, highlighting tumor proximity to ventricular structures. **(D)** Kaplan-Meier OS curves of GB patients according to tumor distance tertiles (T1 = closest, blue; T2 = intermediate, purple; T3 = farthest, red), showing significantly reduced survival in patients with tumors closer to the ventricle. **(E)** Violin plot showing the distribution of tumor distance to the ventricle by surgery type: biopsy (red), partial (purple), and total resection (blue). The plot includes boxplots and individual data points. Statistical analysis using the Kruskal-Wallis test (global *p*-value shown) followed by pairwise Wilcoxon tests with Bonferroni correction revealed no significant differences.

To standardize the classification of tumor proximity to the ventricular system, we used anatomical measurements from coronal brain sections (Figure 3A). Vectors were drawn from the closest tumor edge to the ependymal lining of the lateral ventricle, providing a reproducible method to quantify distance. This schematic was essential for categorizing patients into distance-based groups for survival analyses and ensured consistency in anatomical interpretation across cases. The proximity of the tumor to the ventricular system (Figures 3B,C) also showed a strong and significant association with survival (*p* < 0.0012) (Figure 3D). For Kaplan-Meier survival analysis, patients were divided into three groups according to terciles of the measured distance between the tumor and the ventricular system, resulting in three categories: T1 (closest), T2 (middle), and T3 (farthest) in tertiles. Patients in the closest distance group (T1) had the shortest median OS (3.74 months), followed by those in the intermediate group (T2) with a median OS of 17.48 months. Interestingly, 50% of patients in the farthest group (T3) were still alive at the end of the follow-up period (therefore, median OS was not reached for this group during the study period) (Figure 3E).

To determine whether surgical strategy could act as a confounding factor, the relationship between tumor–ventricle distance and extent of resection was evaluated. No significant differences in tumor–ventricle distance were observed across surgery types (biopsy, partial resection, total resection; Kruskal–Wallis test, *p* = 0.91), indicating that ventricular proximity was independent of the surgical approach and supporting its role as an intrinsic anatomical and biological feature rather than a consequence of surgical strategy.

### Survival analysis, tumor size and relation to distance to the ventricle

**FIGURE 4:**
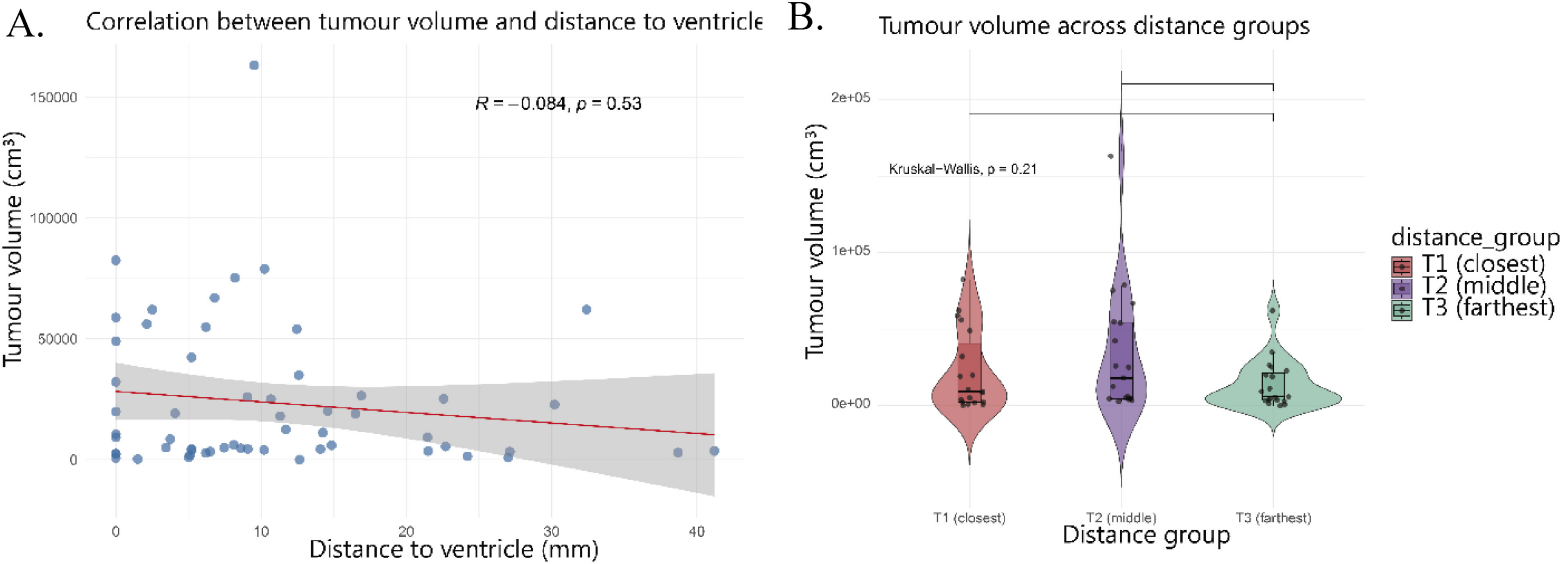
Relationship between tumor volume, ventricular proximity, and survival. **(A)** Correlation between tumor volume (cmł) and distance to the ventricle (mm). No significant association was observed (*p* = 0.53). **(B)** Violin plots showing tumor volume (cmł) across distance tertiles (“Close,” “Middle,” “Far”), with no significant differences detected between groups.

In Figure 4A, tumor volume is plotted against survival time. Kaplan–Meier analysis of tumor volume and survival did not reveal significant differences between tertile groups (*p* = 0.42), confirming that tumor size alone does not determine patient outcome. The direct correlation analysis likewise showed no significant association between tumor size and overall survival (*p* = 0.85). When patients were stratified into tertiles of tumor size (small, medium, large), the violin plot (Figure 4B) suggested a tendency for larger tumors to be associated with shorter survival times; however, this trend did not reach statistical significance (*p* = 0.21). Overall, these results indicate that tumor volume alone does not significantly influence patient survival and is not associated with ventricular proximity, suggesting that spatial tumor characteristics rather than size *per se* are more relevant determinants of outcome.

### Genetic analysis

**FIGURE 5:**
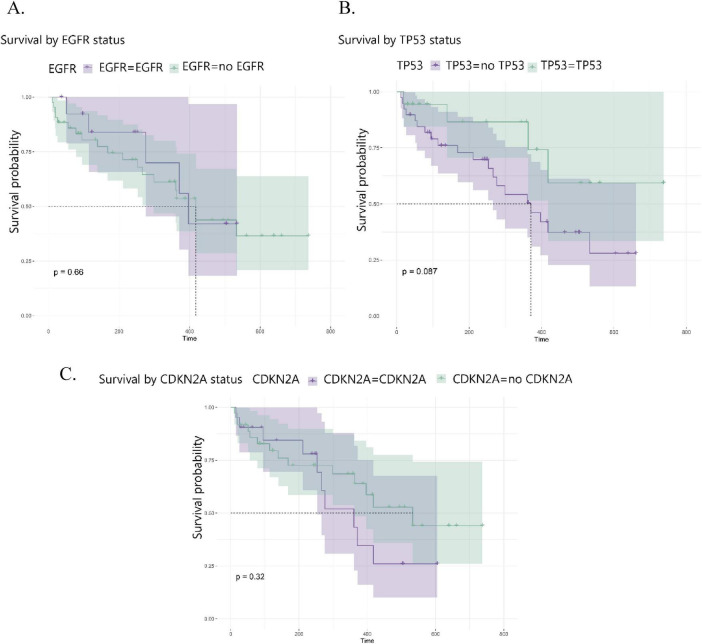
Kaplan-Meier survival curves for GB patients stratified by mutation status of key genes: **(A)** *TP53*, **(B)** *EGFR*, and **(C)** *CDKN2A*. Wild-type and mutated forms shown in green and orange, respectively.

In this study, no statistically significant associations were detected between overall survival or progression-free survival and mutations in the analyzed genes, including *EGFR*, *TP53*, and *CDKN2A*. Similarly, copy number variations (CNVs) affecting *EGFR*, *CDKN2A*, *FGFR2*, and *PTEN* were not significantly associated with survival outcomes within this cohort (Figures 5A–C).

Given the limited sample size, these results should be interpreted with caution, as the absence of statistical significance may reflect limited power rather than the absence of a true biological association. Accordingly, these analyses were conducted for exploratory purposes and are not intended to establish definitive prognostic effects of individual genetic alterations.

While these alterations are well recognized drivers of glioblastoma initiation and progression, their high prevalence across tumors may reduce their ability to discriminate survival outcomes when analyzed in isolation. Moreover, glioblastoma prognosis is increasingly understood to be influenced by non-genetic factors, including tumor–microenvironment interactions, spatial localization within the brain, and proximity to anatomical niches such as the ventricular–subventricular zone.

In this context, genetic alterations may contribute to tumor aggressiveness in combination with spatial and microenvironmental factors, rather than acting as independent determinants of patient survival.

### Lifestyle and medical history

**FIGURE 6:**
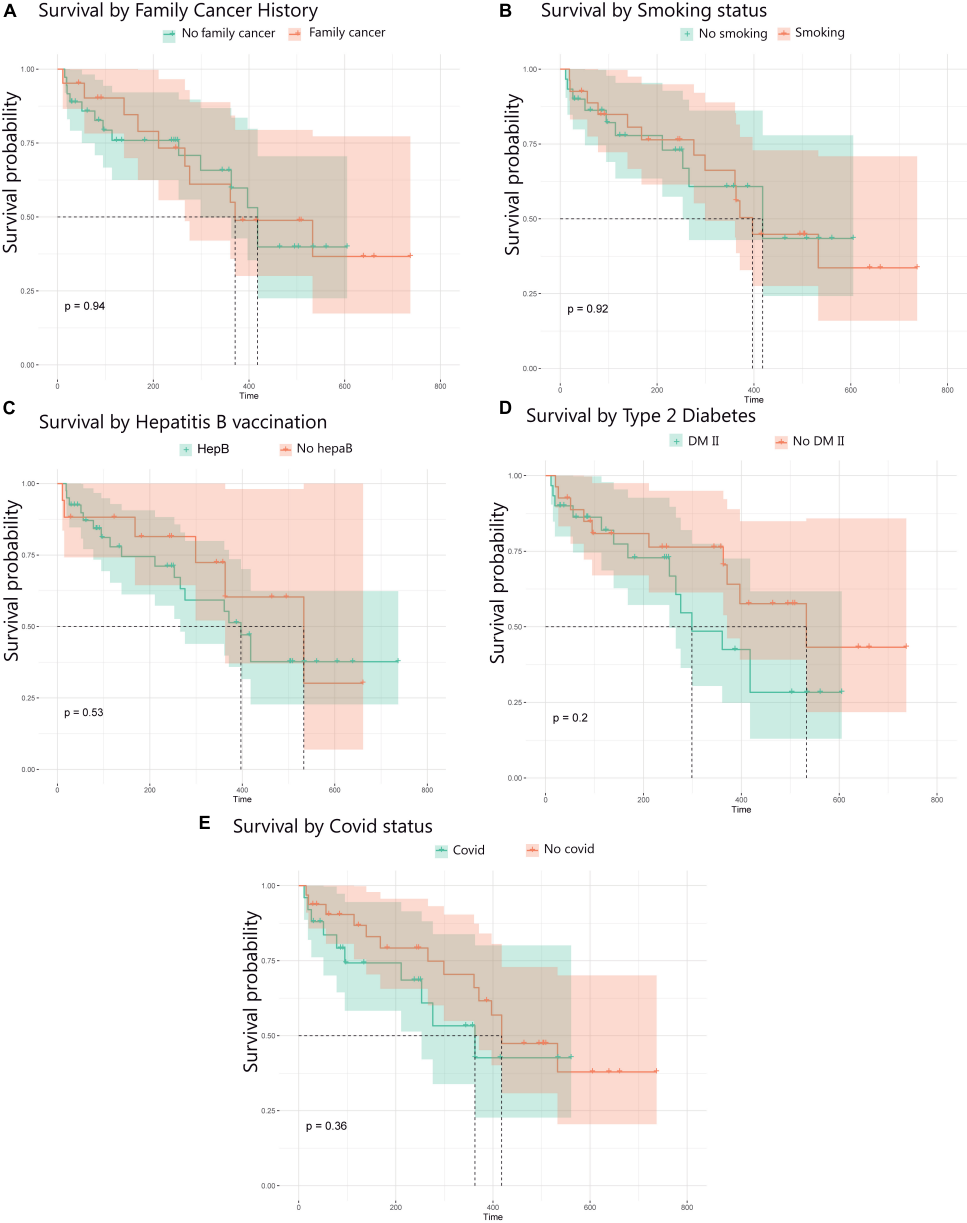
Kaplan–Meier survival analysis for GB patients stratified by lifestyle and medical history. **(A)** Survival stratified by family history of cancer. **(B)** Survival based on smoking status (smokers vs. non-smokers. **(C)** Survival of patients with a history of Hepatitis B. **(D)** Type 2 diabetes. **(E)** Patients with a history of COVID.

Lifestyle and medical history factors, such as smoking habits, previous cancer diagnoses, a history of COVID-19 infection, hepatitis B status, or Type II diabetes, did not show any significant impact on survival outcomes in this group of patients (Figures 6A–E). The analysis of lifestyle and comorbidity variables was exploratory in nature and aimed to identify potential trends rather than establish definitive prognostic associations.

## Discussion

The proximity of the tumor to the lateral ventricle emerged as the most robust prognostic factor. Patients with tumors directly contacting or located closer to the ventricle had markedly shorter survival, whereas those farther away had longer survival. This establishes ventricular proximity as the most robust association observed in this cohort. Importantly, all analyses were restricted to IDH-wildtype glioblastomas, in line with the WHO 2021 classification, thereby avoiding the inclusion of biologically and clinically distinct IDH-mutant astrocytomas.

Surgical resection also showed an effect, though less pronounced than ventricular distance. Patients who underwent gross total resection lived longer than those with subtotal resection or biopsy only, which is consistent with the biological rationale that removing a larger amount of tumor tissue may delay time to recurrence.

However, the distance of the tumor to the ventricles, as well as surgery type, had a strong correlation with survival. Close relationship between ventricular contact and poor outcome raises the hypothesis that cerebrospinal fluid (CSF) dynamics, the neural stem cell niche of the subventricular zone (SVZ), and anatomical structures such as the corpus callosum may contribute to tumor progression and invasiveness. The corpus callosum, a myelin structure that connects the two lateral hemispheres of the brain, is also attached to the lateral ventricle and could act as a conduit for cancer cell invasion. Furthermore, this hypothesis could suggest that a reservoir of neural stem cells is present within the lateral ventricle of the brain, which could act as a reservoir for cancer cells within the tumor. This fact could be assessed by the glioma stem cell marker CD133 (Brescia et al., 2013). Indeed, the SVZ microenvironment has been proposed to foster glioma stem cell maintenance, increasing the likelihood of resistance and recurrence. This biological niche may therefore not only promote initial tumourigenesis but also facilitate repopulation after therapy.

When we examined demographic factors such as age, sex, and BMI, we found that none of them had a significant impact on survival outcomes. This is largely in line with other studies (González Bonet et al., 2022), which suggest that these factors are less critical for predicting overall survival in GB compared to other variables, such as treatment options or tumor behavior (Cabanne et al., 2013), tumor spatial features such as ventricular proximity or surgical extent. As for treatments like radiotherapy and chemotherapy, including the Stupp protocol, they did not seem to significantly impact survival. This could be due to GB complexity, since different patients respond to treatment in very different ways, making it harder to draw clear conclusions. Regarding tumor location, most were in the frontal and temporal lobes. However, surprisingly, specific tumor location did not seem to affect patient survival. Moreover, to rule out the hypothesis that patients undergoing biopsy surgery who tend to have shorter survival rates might have tumors located closer to the ventricle (which would make surgery more difficult), we analyzed the relationship between surgery type and tumor proximity. Our analysis showed no significant association between the distance to the ventricle and the type of surgery performed. This suggests that the poorer survival observed in biopsy patients is not simply explained by tumor location near the ventricle.

This reinforces the idea that the proximity of the tumor to the ventricular system has an intrinsic biological impact on tumor aggressiveness and not merely a mechanical or surgical limitation. Also, our results show no significant association between tumor size and patient survival, despite a non-significant trend toward poorer outcomes with larger tumors. Additionally, tumor volume does not correlate with its distance to the ventricle, indicating size is independent of location. These findings suggest that tumor size and proximity to the ventricle alone are insufficient predictors of prognosis in glioblastoma. Other factors, such as tumor invasiveness or microenvironmental interactions, may have greater influence on patient outcomes. Further research is needed to elucidate these mechanisms and improve prognostic models. Interestingly, while tumor size did not significantly impact survival, tumor proximity to the ventricular system emerged as a strong prognostic factor. This finding suggests that spatial tumor location, reflecting interactions with specific anatomical and biological niches, may be more relevant than tumor burden alone in determining clinical outcome. These results support the concept that glioblastoma aggressiveness is not solely driven by tumor volume but also by its anatomical context and microenvironmental interactions.

Nonetheless, when tumor proximity to the ventricle is combined with other features such as stem cell-like molecular signatures, the predictive value for poor prognosis becomes even more compelling, potentially identifying a more aggressive glioblastoma subtype. Regarding genetic factors, mutations in GB driver genes such as *IDH1*, *EGFR*, *TP53*, and *CDKN2A*, or CNVs in genes like *EGFR* and *PTEN*, did not show strong associations with survival outcomes in this study. However, these findings should be interpreted with caution, as the limited sample size may have reduced the statistical power to detect significant genetic effects. It is likely that the absence of a significant difference in *IDH* mutation is attributable to the limited size of the mutated group. Furthermore, it should be noted that the current classification of GB does not include *IDH* mutation as a diagnostic criterion. While other studies (Campbell et al., 2016; Mao et al., 2012; Verhaak et al., 2010) have found links between certain genetic markers and survival in GB, we did not observe the same results. This could be due to patient differences or because our sample size was too small to pick up on subtle effects, but it is clear that we still lack considerable insight on how genetic changes impact GB.

Several studies have shown no consistent association between glioblastoma and common comorbidities such as smoking (Li et al., 2016), type 2 diabetes (Lu et al., 2018), hepatitis B (Cabanne et al., 2013) infection, or a family history of cancer. These observations should be interpreted cautiously, as the analysis of lifestyle and medical history factors was exploratory and the study was not powered to detect subtle effects of these variables on survival (Qi et al., 2023).

Although glioblastoma recurrence rates of up to 70%–80% have been reported in the literature (Weller et al., 2021), recurrence was documented in 43.9% of patients in our cohort, likely reflecting follow-up duration and censoring. Unfortunately, secondary surgeries for recurrent tumors did not seem to improve survival, which really highlights how limited our treatment options are when GB reappears. We need better strategies for dealing with recurrent GB, something that clearly still is a challenge in the field.

On the other hand, when we looked at lifestyle and medical history factors like smoking, past cancer, COVID-19 infection, and conditions like hepatitis B and Type II diabetes, they did not seem to affect survival at all. This suggests that these factors might not matter as much in the context of GB. However, in GB patients who are motivated to make lifestyle adjustments to improve their outcomes, the exciting news is that dietary restriction of sugar and caloric intake in particular seems to show some promise, with better news coming from exercise, vitamin supplementation, and cannabis use showing potential benefits as well (Peeri et al., 2021; Travers and Litofsky, 2021).

A significant challenge in the management of GB is the difficulty neurosurgeons face in accessing the tumor (Brown et al., 2016; Lacroix et al., 2001; Stummer et al., 2008). The primary objective is to achieve complete removal of the tumor without compromising the patient’s life. However, complete eradication of infiltrating tumor cells is not feasible, as glioblastoma diffusely invades surrounding brain tissue, limiting surgical clearance and contributing to inevitable recurrence (Brown et al., 2016; Lacroix et al., 2001; Stummer et al., 2008). This is a consequence of the high invasiveness of brain cells, which renders total surgery a viable option for increasing survival but not preventing relapse.

What remains evident is that tumors close to the ventricular system present a double challenge: not only are they harder to resect completely due to their location, but they may also represent a biologically distinct and more aggressive subtype of glioblastoma. Perhaps the most relevant finding of this work is that tumors located close to the ventricular system are associated with shorter survival. Patients with tumors that were in direct contact with the ventricles had the shortest survival times, which suggests that where the tumor is located in relation to the ventricles might influence tumor growth or treatment efficacy. This spatial relationship is critical and could serve as a valuable addition to current prognostic models. Classifying tumors by their proximity to the ventricle at diagnosis could help stratify patients more accurately and inform treatment decisions, including whether to consider more aggressive or targeted therapies in periventricular tumors (Mistry et al., 2017, 2020). Notably, the association between ventricular proximity and poor survival could not be explained by differences in surgical strategy, as tumor ventricle distance was independent of the extent of resection. This supports the hypothesis that ventricular proximity reflects intrinsic biological aggressiveness rather than a purely surgical limitation.

Extensive resection has consistently been associated with prolonged survival in glioblastoma patients (Brown et al., 2016; Lacroix et al., 2001; Stummer et al., 2008). While genetic factors did not provide us with clear answers, it is still important to keep studying them, as they could hold the key to better treatments in the future. The relationship between tumor proximity to the ventricle and survival is also something that deserves more attention, as it could help us improve prognostic models. Several studies have reported that glioblastomas contacting the subventricular zone (SVZ) are associated with poorer prognosis and increased likelihood of multifocal recurrence. This may be related to the presence of neural stem-like cells in the SVZ microenvironment, which could contribute to tumor aggressiveness and resistance to treatment. Lateral ventricle glioblastomas may represent a more aggressive and invasive tumor subtype and integrating this spatial parameter into clinical assessment could help personalize therapy and ultimately improve patient outcomes. Overall, there is still a lot we need to figure out, but the hope is that by continuing to explore new treatment options and gathering more data, we can find better ways to help patients with GB in the future. Advancements in immunotherapy, tumor-treating fields, and targeted therapies offer promise, yet their integration into standard treatments remains a work in progress, highlighting the need for comprehensive, individualized treatment strategies (Qi et al., 2023; Weller et al., 2021). Given the single-center retrospective design and cohort-specific tertile cut-offs, these findings should be interpreted as exploratory and require validation in independent cohorts.

## Data Availability

The original contributions presented in the study are included in the article/Supplementary material, further inquiries can be directed to the corresponding author/s.
